# Linearly integrating speed and accuracy to measure individual differences in theory of mind: Evidence from autistic and neurotypical adults

**DOI:** 10.1177/17470218231165251

**Published:** 2023-05-18

**Authors:** Lucy Anne Livingston, Punit Shah, Francesca Happé

**Affiliations:** 1 Department of Psychology, Institute of Psychiatry, Psychology and Neuroscience, King’s College London, London, UK; 2 Neuroscience and Mental Health Innovation Institute, Cardiff University, Cardiff, UK; 3 Department of Psychology, University of Bath, Bath, UK; 4 Social, Genetic and Developmental Psychiatry Centre, Institute of Psychiatry, Psychology, & Neuroscience, King’s College London, London, UK

**Keywords:** Theory of mind, social cognition, autism, adults, response time, accuracy

## Abstract

It has long been theorised that there is a direct link between individual differences in social cognition and behaviour. One of the most popular tests of this theory has involved examination of Theory of Mind (ToM) difficulties in Autism Spectrum Disorder (ASD). However, evidence for associations between ToM and social behaviour is mixed, both when testing the ToM explanation of ASD and when investigating individual differences in ToM in the general population. We argue that this is due to methodological limitations of many ToM measures, such as a lack of variability in task performance, inappropriate non-ToM control tasks, and a failure to account for general mental ability. To overcome these issues, we designed a novel task, which probed individual differences in ToM fluency through mental state attribution in response to cartoons (Cartoons Theory of Mind [CarToM] task). This task, enabling the linear combination of speed and accuracy, was used to quantify ToM ability and its association with self-reported (a)typical social behaviour in adults with and without ASD. In a large sample (*N* = 237), we found that having an ASD diagnosis and higher autistic traits predicted lower ToM ability, even after accounting for performance on a well-matched non-ToM condition and general mental ability. Overall, our findings provide fresh support for the existence of a link between individual differences in social cognition (specifically, ToM) and behaviour (specifically, autism). This has implications for social-cognitive theory and research, allowing large-scale, online assessment of individual differences in ToM in clinical groups and the general population.

## Introduction

“Theory of mind” (ToM)—the ability to attribute mental states (e.g., beliefs, desires) to predict and explain others’ behaviour (Baron-Cohen et al., 2000)—is thought to be a fundamental component of social cognition that underpins a range of adaptive social behaviours (Happé et al., 2017). However, evidence in support of this theoretical view is mixed and warrants further research. One powerful way to explore the association between social cognition and behaviour involves studying Autism Spectrum Disorder (ASD; Livingston & Happé, 2021), a condition characterised by atypical social behaviour (American Psychiatric Association, 2013). Meta-analyses suggest that individuals with ASD demonstrate ToM difficulties compared with their neurotypical counterparts, reflecting some of the largest group differences of any cognitive domain (Bottema-Beutel et al., 2019; Velikonja et al., 2019). However, inconsistencies do exist. Some studies report clear ToM difficulties in autistic compared with non-autistic individuals (e.g., Chung et al., 2014; Morrison et al., 2019) and others find small (if any) group differences (e.g., Brewer et al., 2017; Lever & Geurts, 2016). Even when ToM difficulties are found in autism, while ToM scores correlate with observable social impairments and functioning in some studies (e.g., Jones et al., 2018), this is not always the case ToM scores do not necessarily correlate with observable social impairments and functioning (see Brunsdon & Happé, 2014; Sasson et al., 2020). Furthermore, in neurotypical samples, ToM task performance has rarely been found to be linked to real-world social skills (e.g., social network size; although see Stiller & Dunbar, 2007), even in the most recent research (see Qureshi et al., 2020).

Contrary to popular theory, then, the relationship between ToM task performance and real-world social dis/abilities is not as clear as might be expected. On the one hand, it is possible that there is only a very weak link between individual differences in social cognition and behaviour. On the other hand, there are numerous methodological issues with current social-cognitive (e.g., ToM) tasks, which may be stymying theoretical and clinical understanding of links between (a/typical) social cognition and behaviour (Livingston, Carr & Shah, 2019). Therefore, in this study, we explored and addressed several methodological issues to directly inform a novel empirical investigation of the link between individual differences in social cognition, specifically ToM, and a/typical social behaviour, specifically autism/autistic traits.

First, many popular tasks, originally designed for children, suffer from ceiling effects when applied to adult populations, thus failing to capture the full range of individual differences. For example, White and colleagues (2009) found that while the Strange Stories Task (Happé, 1994)—in which participants infer speakers’ mental states from written vignettes—differentiated autistic from neurotypical children, both autistic and neurotypical adults showed extremely high accuracy, and there was only a “trend-level” group difference. Other tasks may not capture variability in adults due to a limited range of possible scores. For example, the Frith–Happé Animations Test, successfully employed in child and adolescent populations (e.g., Jones et al., 2018), has scores that range from 0 to 8 when responses are given spontaneously (Abell et al., 2000) or 0 to 4 in the multiple-choice version of the task (Livingston et al., 2021; White et al., 2011). This limited range makes it challenging to study how individual differences in ToM in adults relate to real-world social ability.

Second, some ToM tasks do not specifically isolate ToM, independent of intelligence, memory, attention, or other social-emotional processes. For example, performance on the Reading the Mind in the Eyes Test (RMET; Baron-Cohen, Wheelwright, Hill, et al., 2001), which requires participants to infer mental states from photos of the eye region, correlates highly with verbal ability (Baker et al., 2014) and education level (Dodell-Feder et al., 2014). While there is a “control” version of the RMET, where participants are required to infer gender from images of eyes, it is rarely employed in research as it produces ceiling effects. More generally, matching non-ToM “control” conditions to ToM conditions in terms of cognitive complexity is challenging (see White et al., 2009), particularly given the specific cognitive demand in ToM tasks to suppress one’s own representation of the world to adopt that of another (see Apperly, 2012). We propose that addressing this issue requires a carefully matched non-ToM control condition *and* administration of other cognitive measures (e.g., measuring general mental ability). Together, these measures would permit analyses controlling for *both* performance on the non-ToM control task and general mental ability to ensure that any association between ToM and social behaviour is specific to ToM. This approach is rarely taken in autism research, or ToM research more generally (although see Brewer et al., 2017). This is perhaps due to lack of variance in ToM scores and/or underpowered studies where the shared variance between ToM and non-ToM conditions is so large that any unique association between ToM and social behaviour, after accounting for non-ToM performance, cannot be detected.

Third, and central to this study, it is now recognised that some people with social difficulties can use “compensatory strategies” to circumvent their underlying ToM difficulties (Livingston & Happé, 2017; Livingston, Shah, & Happé, 2019). For example, they may rely on intelligence (i.e., a non-ToM route) to logically “work out” the correct answer in ToM tasks. Critically, almost all current ToM tasks take accuracy to index “good ToM” without imposing a time limit on participants to complete each task trial. This means that, given enough time to engage compensatory mechanisms, neurotypical adults with subtle ToM difficulties, or even autistic adults with more significant difficulties, can perform well on ToM tasks (see Livingston, Colvert, et al., 2019). Arguably, implicit measures of ToM, such as anticipatory looking behaviour during false belief tests, help to circumvent this issue of compensatory strategies. Indeed, Senju et al. (2009) showed that autistic adults can solve explicit false belief tests, but do not spontaneously track the false belief of a protagonist as evidenced by anticipatory looking, thus suggesting implicit ToM difficulties despite good explicit ToM. However, the extent to which implicit and explicit ToM reflect two distinct mechanisms (e.g., Apperly & Butterfill, 2009) or two expressions of the same mechanism at different points in development (e.g., Low & Perner, 2012), or whether implicit ToM exists at all (see Kulke et al., 2018) remains highly debated. Moreover, measuring so-called implicit ToM is often time-consuming and requires technical equipment (e.g., eye tracking), making implicit ToM measures unviable for large-scale studies of individual differences in social cognition.

Finally, it has been argued that most current ToM tasks lack ecological validity; they do not reflect ToM use in the real world. There have been developments in recent years with the introduction of video-based ToM tasks (e.g., Baksh et al., 2020; Brewer et al., 2017; Dziobek et al., 2006; Murray et al., 2017), which require participants to infer characters’ mental states in dynamic interactions, alongside control and/or memory questions. One study suggests that these video-based measures may better capture variance in adults with varying autistic traits compared with previous tasks (e.g., Stewart et al., 2020). However, it is possible that these more naturalistic ToM tasks may also be solved via compensatory mechanisms if participants are given sufficient time to answer mental state questions. Individuals, particularly those with ASD, may be able to “hack out” the correct answer by using (slower) strategies over the course of watching a video clip. Therefore, video-based tasks may still not measure true ToM ability. Indeed, in the real world, good social ability requires inferences about others’ mental states that are not only *accurate* but also sufficiently *fast* to facilitate an appropriate response in a dynamic social interaction. In addition, video-based tasks are time-consuming to administer (e.g., the popular Movie for the Assessment of Social Cognition [Dziobek et al., 2006] takes ~40 mins) and require coding of participants’ qualitative responses. Hence, their use in large-scale online studies (e.g., genetically sensitive population-based studies), required to address key questions about how individual differences in ToM are linked to a range of socially relevant phenomena, is limited (see also Livingston, Carr, & Shah, 2019). While their continued development is important, so too is the development of shorter ToM measures that are less susceptible to hacking via compensation and possible to administer in large-scale studies for well-powered statistical analyses.

### The current study

Taken together, there are several issues with previous tasks that are stymying advances in our theoretical understanding of individual differences in ToM ability and links with autism. To address these issues, we suggest that measurement of ToM in adults will be facilitated by methods that capture sufficient variability in task performance, include control stimuli that are as closely matched as possible to ToM stimuli, take account of speed of processing, and enable multivariate statistical analyses in which ToM can be quantified independently of other cognitive processes (e.g., general mental ability). Therefore, building upon a concept and stimuli developed by Corcoran et al. (1997) and Happé and colleagues (1999; Gallagher et al., 2000), we developed a new web-based Cartoons Theory of Mind (CarToM) task that probes mental state understanding of humorous cartoons, alongside well-matched non-ToM stimuli. Importantly, this task enabled collection of response time (RT) data, which are frequently used to quantify individual differences in cognitive processing, but are arguably under-used in autism and social cognition research. RT data also enable participants’ potential use of compensatory processing to be detected. For example, it is likely that non-ToM routes or strategies to achieving “good” ToM performance are slower and more deliberative than typical routes via the ToM network (Livingston & Happé, 2017; White et al., 2014). This should be reflected in longer RTs, which can then be included in analyses. More specifically, using novel analytic methods for the first time in this field of research, we linearly integrated RT and accuracy data to measure task performance (Vandierendonck, 2017). Furthermore, we recruited a sufficiently large sample of people with and without ASD, who all completed a measure of general mental ability, enabling multivariate analyses to test for the unique link between ToM ability and autism over and above general mental ability and performance on the non-ToM trials.

Following the ToM theory of ASD (see Livingston & Happé, 2021), we hypothesised that autism (both an autism diagnosis and higher autistic traits, irrespective of diagnosis), would be uniquely associated with poorer ToM task performance, even after controlling for non-ToM performance and general mental ability. Conversely, we predicted that non-ToM performance would not be associated with ASD status or autistic traits after accounting for ToM performance and general mental ability.

## Methods

### Participants

Participants were 237 adults (116 females) aged 18–80 years. Seventy-two participants reported a clinical autism diagnosis (ASD group; 18–67 years) and 165 individuals reported no diagnosed neurological, psychiatric, or neurodevelopmental conditions (neurotypical group; 18–80 years). All participants were recruited via online sources (e.g., social media, word of mouth, university participant pools). Autistic participants were additionally recruited via the UK charity, the *National Autistic Society*, by advertising the study to adults accessing their services. Autistic participants had a confirmed autism diagnosis and supplied information about the type of healthcare professional(s) who made their diagnosis. All participants had normal or corrected-to-normal vision and gave informed consent. Ethical permission was obtained from the Psychiatry, Nursing, and Midwifery Research Ethics Subcommittee at King’s College London. G*Power analysis (Faul et al., 2007) suggested that, given our sample size, we would have 80% power to detect small-to-medium effects (*f*^2^ = 0.03, α = 0.05, two-tailed) in our regression analyses.

### Cartoons theory of mind task

**Figure 1.**
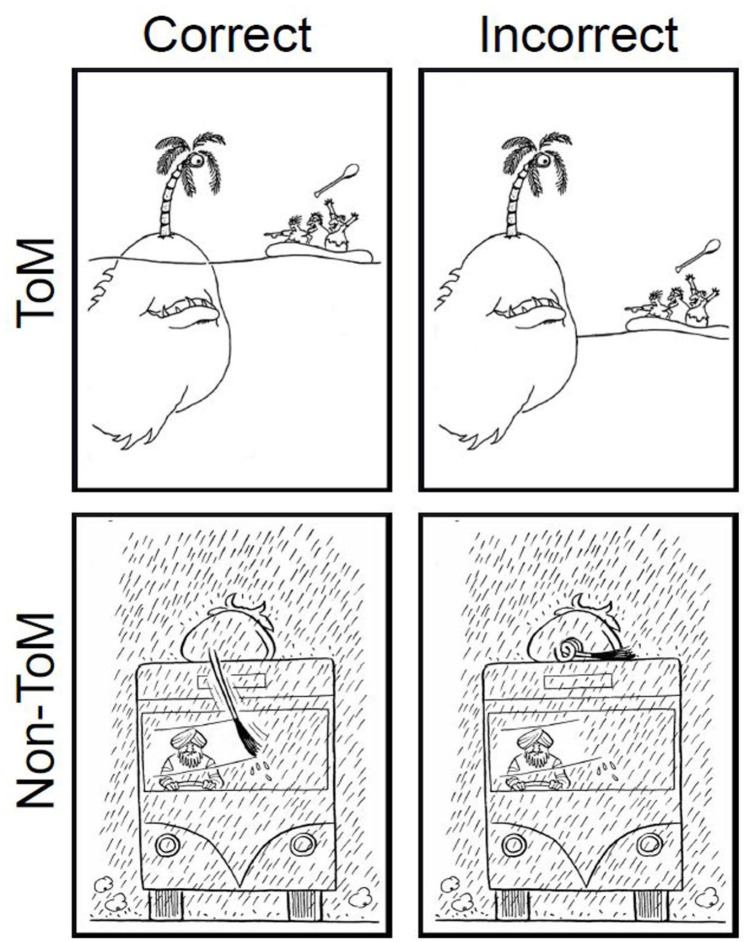
Example stimuli for the CarToM task. *Note.* Example pairs of ToM cartoons (top) and non-ToM cartoons (bottom) from the CarToM task. The original and humorous cartoon (left) in each pair is the “correct” answer. The cartoon edited to be less/no longer humorous (right) in each pair is the “incorrect” answer.

Stimuli were 28 pairs of cartoons, sourced from online material, books, and magazines (e.g., The New Yorker). Following Happé et al. (1999), cartoons were chosen for their humour, due to either ToM reasoning (e.g., a character has a false belief; ToM condition) or physical reasoning (e.g., a physical impossibility, slapstick comedy; non-ToM condition). In each cartoon pair, there was an “original” and an almost identical “edited” version with a minor visual change to remove its humour (see Figure 1). Importantly, these changes did not significantly alter visual complexity (measured in terms of greyscale entropy; see Gonzalez et al., 2004) between original and edited cartoons (*Z* = 226.50, *p* = .60, *r* = .12^1^). In total, we developed 14 ToM and 14 non-ToM cartoon pairs, which also did not significantly differ in visual complexity (*U* = 118.00, *p* = .38, *r* = .20).^1^ Cartoon pairs were presented simultaneously on screen using a spatial two-alternative forced choice (2AFC) design, and each trial was separated by a 500-ms inter-stimulus interval (ISI) of a central fixation cross. Cartoons remained on screen until participants selected the cartoon they found most humorous using the mouse. The “correct” cartoon was the original and the edited version was the “incorrect” answer. The order of cartoon pairs, and side of the screen on which the “correct” cartoon in each pair appeared, was randomised across participants. The mouse was reset to the central fixation cross at the beginning of each trial, which also helped to ensure that the RT was measured from the onset of the images that were immediately presented following the fixation cross. Participants completed three practice trials to familiarise them with the procedure, followed by 28 experimental trials. They received no feedback in the practice or experimental trials.

Percentage accuracy and mean RTs (for correct trials) were calculated for ToM and non-ToM conditions separately. Trials with RTs ± 3 *SD*s of each participant’s overall mean RT were excluded. This resulted in a small percentage of trials being excluded (*M* = 1.42%, *SD* = 1.78%), with no significant difference between the ASD (*M* = 1.39%, *SD* = 1.85%) and neurotypical (*M* = 1.43%, *SD* = 1.76%) groups, *t*(235) = 0.16, *p* = .88, *d* = 0.02. Accuracy and RT data were combined using the Linear Integrated Speed–Accuracy Score (LISAS) method (Vandierendonck, 2017). This method was chosen over other speed–accuracy combination methods (e.g., Inverse Efficiency Scores [IES], Rate Correct Score [RCS]), as it has been shown to be one of the most robust; for example, RCS can produce spurious effects and IES is only valid when error rates are very low (see Vandierendonck, 2017). LISAS scores form the critical measure of ToM ability in this study as follows:

$$\mathrm{LISAS}\;=RT_{ij}+PE_{ij}\times \frac{S_{r}}{S_{e}}$$

where *RT*_ij_ and *PE*_ij_ refer to the mean RT on correct trials and proportion error [1−(percentage accuracy/100)], respectively, for participant *i* in condition *j* (i.e., ToM or non-ToM). *S_r_* and *S_e_* refer to the standard deviation for RT and PE, respectively, calculated using participant *i*’s total correct RT and PE across both conditions. Together, this computation ensures that RT and PE are weighted equally in the LISAS scores. Final ToM and non-ToM scores are interpreted as mean correct RT (ms) corrected for the number of errors made. Therefore, overall greater scores reflect poorer task performance.

Addressing the previously outlined issues with ToM measures for a/typical adults, (1) the CarToM is designed to capture variability without ceiling effects using LISAS and stimuli are specifically designed for adults; (2) non-ToM and ToM conditions are closely matched on task demands and low-level visual properties, and the task generally has low verbal and memory demands; (3) continuous LISAS data are particularly suitable for multivariate analyses; (4) the task does not require explicit mentalising, and therefore is less likely to prompt participants’ use of deliberate compensatory strategies compared with previous tasks; and finally, (5) the task is short, with objective and automated scoring protocol.

### Other cognitive tasks and questionnaires

#### Autistic traits

Autistic traits were quantified using the 50-item Autism-Quotient (AQ; Baron-Cohen, Wheelwright, Skinner, et al., 2001). The AQ is widely used in autistic and non-autistic populations, and scores can vary from 0 to 50. In particular, the AQ can be used to perform powerful trait-wise analyses irrespective of autism status (e.g., Shah et al., 2019). In this study, the AQ had good internal reliability (ASD group: *a* = .86, neurotypical group: *a* = .81).

#### General mental ability

General mental ability was estimated using the Spot the Word task (STW; Baddeley et al., 1993), which has demonstrated convergent validity with the Wechsler Adult Intelligence Scale (Yuspeh & Vanderploeg, 2000). Word pairs, comprising a real word (e.g., albatross) and non-word (e.g., zando), were simultaneously presented on screen. Participants were tasked with identifying the real word as quickly and accurately as possible using their mouse. They completed three practice trials before 60 experimental trials. Importantly, the general procedure (e.g., 2AFC format, ISI duration) closely matched the CarToM. Indeed, the STW task was chosen over other general mental ability/intelligence measures because it was possible to match it to the general task demands and the LISAS approach to measure performance on the CarToM. Accordingly, performance combined speed and accuracy, such that lower scores are indicative of better performance (i.e., faster performance while accounting for accuracy). A small percentage of trials were excluded (*M* = 1.00%, *SD* = 0.66), following the criteria used for the CarToM, and did not significantly differ between the two groups, *t*(113.35) = −0.26, *p* = .80, *d* = 0.04.

#### Other ToM tasks

Participants completed two additional ToM tasks, which have previously been administered online (e.g., Germine et al., 2012; Livingston et al., 2021). The Reading the Mind in the Eyes Test (RMET; Baron-Cohen, Wheelwright, Hill, et al., 2001) requires participants to view images of the eye region and choose the word that best describes the emotional state being depicted, from a choice of four words. Participants completed one practice before 36 experimental trials. In the Frith–Happé Animations Test (Animations Test; Livingston et al., 2021; White et al., 2011), participants watch 12 videos of two triangles interacting. The triangles move randomly (e.g., drifting; “Random”), interact in a goal-directed manner (e.g., fighting; “Goal-directed”), or one triangle is manipulating the other’s mental state (e.g., tricking; “ToM”). After presentation of each video, participants choose whether the interaction was random, goal-directed, or mental using a mouse response. Participants completed three practice trials (one for each condition) before the 12 experimental trials. Trials in both the RMET and Animations Test were presented in a randomised order and no feedback was provided.

The study was built using the online experiment builder *Gorilla* (Gorilla.sc; Anwyl- et al., 2020), which has been validated for the selection of stimuli and collection of RT data via mouse press (Anwyl-Irvine et al., 2021). Participants accessed the study remotely via a web browser on their own computer. They first completed demographic information about their age and sex (male, female) and the AQ questionnaire, followed by the cognitive tasks in a randomised order. The procedure was completed on different browsers, at different resolutions, in line with previous web-based research (Anwyl-Irvine et al., 2020).

## Results

Across all participants, speed–accuracy scores (i.e., LISAS)—where greater scores reflect poorer performance—ranged widely for both ToM (*M* = 9,753 ms, *SD* = 3,183 ms) and non-ToM (*M* = 8,623 ms, *SD* = 2,812 ms) conditions, suggesting good variability in task performance and no ceiling effect (see Supplementary Figure 1 for distributions). In addition, performance on the CarToM was generally high, suggesting good engagement with the task (see Supplementary Table 1 for LISAS, accuracy, and RT data). Internal reliability was also acceptable for both ToM (split-half = .68) and non-ToM (split-half = .62) conditions. Preliminary analyses showed that neither ToM nor non-ToM scores differed by sex (*p*s ⩾ .67, *d*s ⩽ 0.06), but greater age (ToM: *r* = .17, *p* = .008, non-ToM: *r* = .17, *p* = .009) and general mental ability (ToM: *r* = .18, *p* = .005, non-ToM: *r* = .20, *p* = .003) were associated with better performance. Having more autistic traits was linked to poorer ToM (*r* = .20, *p* = .002), but not non-ToM (*r* = .08, *p* = .20) scores, with the former correlation being significantly larger than the latter (*z* = −2.89, *p* = .004). See Supplementary Table 2 for correlations among all measured variables.

Table 1 shows participant characteristics by group. ASD and neurotypical groups did not differ significantly in age, general mental ability, or sex, but as expected, autistic participants reported significantly greater autistic traits than neurotypical participants. To determine the unique contribution of group (i.e., ASD vs neurotypical) to ToM scores while controlling for non-ToM scores, general mental ability, age, and sex, data were submitted to multiple linear regression. Having an ASD diagnosis uniquely predicted poorer ToM performance, in line with our hypothesis (Table 2, 1a). Multiple regression also confirmed that, collapsing across groups, autistic traits uniquely predicted poorer ToM performance while accounting for the same covariates (Table 2, 1b). Given that non-ToM scores uniquely predicted ToM scores, in both group- and trait-wise analyses, we performed equivalent regression analyses with non-ToM scores as the outcome variable. However, as predicted, neither having an ASD diagnosis nor autistic traits were a significant unique predictor of non-ToM scores, over and above ToM, general mental ability, age, and sex (Table 2, 2a and 2b). Finally, as the AQ captures both social and non-social features, we re-conducted correlational and regression analyses related to autistic traits, including only the 30 items of the 50-item measure that directly tap social behaviour. The same pattern of significant results was found.

**Table 1.** ASD and neurotypical group characteristics.

	ASD (*n* = 72)	Neurotypical (*n* = 165)	Group comparison
	*M* (*SD*)	*M* (*SD*)	
Age	31.76(11.67)	28.80(12.07)	*t*(235) = −1.76 *p* = .08 *d* = 0.25
Autistic traits	36.35(7.63)	17.44(6.96)	*t*(235) = −18.68 *p* < .001 *d* = 2.64
General mental ability^a^	2243(1203)	2375(1788)	*t*(235) = 0.57 *p* = .57 *d* = 0.08
*n* Male, female	36, 36	85, 80	χ^2^(1) *=* 0.05 *p* = .83 Φ = 0.01

RT: response time.

Autistic traits were measured using the Autism-Quotient (Baron-Cohen, Wheelwright, Skinner, et al., 2001). General mental ability was estimated using the Spot the Word task (Baddeley et al., 1993). Effect sizes are reported as Cohen’s *d* for *t* tests and Phi (Φ) for chi-squared tests.

a Linear Integrated Speed-Accuracy Scores (Vandierendonck, 2017); greater scores reflect longer RTs (ms), corrected for accuracy, that is, poorer performance.

**Table 2.** Regression analyses—CarToM: ToM predicted by (1a) group and (1b) autistic traits, and CarToM: non-ToM predicted by (2a) group and (2b) autistic traits.

(1a) CarToM: ToM—overall model fit: *F*(5, 231) = 80.08, *R*^2^ = .63, *p* < .001							
Predictor	*B*	*SE B*		β		*t*	*p*
Group (1 = ASD, 0 = Neurotypical)	772.66	280.65		0.11		2.75	.006
CarToM: non-ToM	0.86	0.05		0.76		18.29	<.001
General mental ability	0.07	0.08		0.04		0.91	.36
Age	5.26	11.08		0.02		0.47	.64
Sex (1 = male, 0 = female)	211.29	259.71		0.03		0.81	.42
(1b) CarToM: ToM—overall model fit: *F*(5, 231) = 80.27, *R*^2^ = .64, *p* < .001							
Autistic traits	32.42	11.51		0.12		2.82	.005
CarToM: non-ToM	0.87	0.05		0.77		18.71	<.001
General mental ability	0.07	0.08		0.04		0.86	.40
Age	2.99	11.18		0.01		0.27	.79
Sex (1 = male, 0 = female)	138.43	259.86		0.02		0.53	.60
(2a) CarToM: non-ToM—overall model fit: *F*(5, 231) = 76.92, *R*^2^ = .63, *p* < .001							
Predictor	*B*		*SE B*		β	*t*	*p*
Group (1 = ASD, 0 = Neurotypical)	−158.49		254.98		−0.03	−0.62	.54
CarToM: ToM	0.69		0.04		0.78	18.29	<.001
General mental ability	0.08		0.07		0.04	1.04	.30
Age	8.93		9.90		0.04	0.90	.37
Sex (1 = male, 0 = female)	−222.11		232.25		-0.04	-0.96	.34
(2b) CarToM: non-ToM—overall model fit: *F*(5, 231) = 77.94, *R*^2^ = .63, *p* < .001							
Autistic traits	−15.81		10.39		−0.06	−1.52	.13
CarToM: ToM	0.69		0.04		0.78	18.71	<.001
General mental ability	0.07		0.07		0.04	0.98	.33
Age	10.78		9.95		0.05	1.08	.28
Sex (1 = male, 0 = female)	−194.11		231.50		−0.04	-0.84	.40

CarToM: Cartoons Theory of Mind task; ASD: Autism Spectrum Disorder; ToM: Theory of Mind; *B*: unstandardised regression coefficient, *SE*: standard error, β: Standardised regression coefficient; VIF: variance inflation factor.

Greater ToM and non-ToM scores reflect longer RTs (ms), corrected for accuracy, that is, poorer performance. All VIF values were <10, suggesting multicollinearity was not a concern. The residuals were normally distributed and there was no evidence of homoscedasticity. Durbin-Watson values were all ~2, suggesting errors were independent. An equivalent pattern of significant results was found when only the 30 items of the 50-item AQ that directly tap social behaviour were used to measure autistic traits.

Exploratory partial correlations were conducted to explore the relationships between performance on the CarToM, RMET, and Animations Test while controlling for general mental ability (see Table 3). There were small but significant correlations between CarToM ToM scores and performance on the RMET, and the “ToM”, but not “Random” or “Goal-directed,” conditions of the Animations Test. CarToM non-ToM scores did not correlate with performance on either ToM task, after controlling for general mental ability. Means for the RMET and Animations Test by group can be found in Supplementary Table 1.

**Table 3.** Correlations between CarToM, RMET, and Animations Test performance.

	1	2	3	4	5	6
1. CarToM: ToM (LISAS)	—	.78***	−.15*	−.13*	−.08	.08
2. CarToM: Non-ToM (LISAS)	.79***	—	−.12	−.06	−.06	.02
3. RMET (%)	−.16*	−.13*	−	.19^**^	.12	.08
4. Animations—ToM (%)	−.14*	−.06	.19**	—	.26***	.24***
5. Animations—goal-directed (%)	−.08	−.06	.12	.26***	—	.33***
6. Animations—random (%)	−.08	.02	.08	.24***	.33***	—

*Note.* RMET: Reading the Mind in the Eyes Test (Baron-Cohen, Wheelwright, Hill, et al., 2001); Animations: Frith–Happé Animations Test (White et al., 2011); ToM: Theory of Mind; LISAS: Linear Integrated Speed-Accuracy Scores; RT: response time.

Correlations above the diagonal are partial, controlling for general mental ability, and correlations below the diagonal are not. Variables 1–2 are LISAS (Vandierendonck, 2017); greater scores reflect longer RTs (ms), corrected for accuracy, that is, poorer performance. Variables 3–6 are percentage accuracy.

*** *p* < .001, **p < .01, *p < .05.

## Discussion

This study aimed to measure individual differences in ToM ability in adults by employing innovative speed–accuracy scores and using a novel experimental task (the CarToM). This represents a substantial methodological improvement on previous studies. Specifically, we aimed to test the theoretical link between ToM ability and social behaviour by studying atypical ToM in relation to autism. A robust pattern of results emerged. In line with our predictions, poorer ToM task performance was uniquely linked to diagnosed ASD and autistic traits, over and above general mental ability, age, sex, and critically, performance on a closely matched non-ToM condition. Non-ToM performance showed no such relationship with autism or autistic traits, further highlighting the specificity of this effect. These findings indicate that, when using appropriate and sensitive methods to quantify individual differences in ToM, a relationship between ToM and social skills/difficulties in adults is found.

Our findings fundamentally contribute to theories of social cognition. ToM ability has long been theorised to underpin real-world social abilities and behaviours (e.g., Happé et al., 2017), yet evidence for this has been equivocal, particularly in adulthood. This study, however, demonstrated a link between ToM ability and a/typical social behaviour, at least in terms of autism. More specifically, our data speak against proposals that ToM is, on average, unaffected in autism (e.g., Scheeren et al., 2013), and instead support notions that atypical ToM is a useful marker of both diagnosed ASD (e.g., Cantio et al., 2018) and elevated autistic traits in the general population (e.g., Stewart et al., 2020). In addition, the data provide further insight into why ToM may be atypical in relation to autism, which were indicative of slower correct mental state attributions leading to lower ToM ability. Importantly, this finding cannot be explained by generally slower processing in autism (e.g., Haigh et al., 2018), as the link between autism and poorer ToM ability remained after controlling for non-ToM performance. Instead, the reduced speed–accuracy scores for ToM cartoons may be due to a less specialised, mature, fluent, and/or efficient neurocognitive system sub-serving mental state attribution (see White et al., 2014). In addition, or alternatively, autistic participants may have been using slower compensatory mechanisms to complete ToM trials, for example, engaging executive functions (Livingston, Colvert, et al., 2019) and/or semantic memory systems (Ullman & Pullman, 2015). Notwithstanding this outstanding question, which will require neuroimaging methods to address, the present findings suggest that the measurement of ToM ability remains a fruitful research strategy for delineating mechanisms underpinning typical and atypical social behaviour (Happé & Frith, 2014) and is particularly powerful using an RT-sensitive approach.

The relatively clear-cut nature of our findings may be attributable to a number of methodological advantages of the CarToM over previous methods. First, by using combined speed–accuracy scores (rather than accuracy alone) to measure task performance, the CarToM captures normally distributed variability in ToM ability, enabling more fine-grained investigation of individual differences in the fluency of mental state attribution. Indeed, there was no evidence of ceiling effects in either the neurotypical or ASD group. In addition, speed–accuracy scores penalise participants who may be using compensatory strategies to solve ToM, and therefore, the task may be a superior reflection of genuine ToM ability. Second, the CarToM was designed to isolate ToM ability specifically. Indeed, the task does not require participants to process language, give a verbal response, or hold information in memory, thus minimising extraneous task demands present in many previous ToM tasks. This is particularly critical if ToM tasks are to be suitable for testing heterogeneous populations where other cognitive abilities may be affected (e.g., older adults). Promisingly, general mental ability did not significantly predict either ToM or non-ToM performance, once performance on the other condition was accounted for, suggesting that the CarToM is not measuring general mental ability or processing speed. Finally, the CarToM has particularly well-matched non-ToM trials (the same task demands and low-level visual complexity) which, unlike many other studies, enabled us to statistically account for non-ToM performance in regression analyses. The specific relationship between poorer ToM and autism therefore may have been much more tractable than in previous studies for this reason. Given these numerous methodological improvements, it is hoped that the CarToM holds promise for demonstrating links with real-world social abilities in autistic and neurotypical populations, where previous studies have found limited associations (e.g., Sasson et al., 2020). On a final note, while links between ToM performance and performance on the RMET and Animations Test provide some construct validity for the CarToM, the relatively weak strength of these is unsurprising, given the aforementioned methodological distinctions between these tasks (see also Olderbak et al., 2019).

The present findings also have a number of implications for research. First, despite theoretical (Livingston & Happé, 2017) and empirical (Livingston, Colvert, et al., 2019; Livingston, Shah, et al., 2019) evidence for the concept of compensation—that is, apparently good social skills despite poor social cognition—in people with diagnosed ASD and/or high autistic traits, measuring compensation requires a highly sensitive social-cognitive task that cannot be solved via non-social routes. Moving forward, it will be possible to test whether the CarToM can detect ToM difficulties in individuals who heavily compensate in social situations, for example, using self-report measures of compensation (Hull et al., 2019; Livingston et al., 2020). Second, given that the CarToM is short, administered online, and does not require subjective scoring, our new task is suitable for use in large-scale studies to address crucial questions about ToM ability and individual differences in several domains (e.g., personality, mental health, genetics). The task may also serve a function in future neuroimaging studies aimed at delineating the neurocognitive mechanisms of ToM, as well as compensatory neurocognitive routes underlying “good” ToM task performance (see Livingston & Happé, 2017; White et al., 2014). The forced-choice design of the task makes it particularly amenable to neuroimaging methods. Finally, the results highlight the potential utility of using speed–accuracy scores in social-cognitive science more generally, in particular where researchers wish to account for compensatory strategies or alternative routes to achieve similar levels of task performance. However, we do recognise that not every social-cognitive task will lend itself to the LISAS approach, for example, if it can be assumed that RT and percentage error have different origins (see Vandierendonck, 2017, for detailed discussion).

Our findings should be interpreted in light of some limitations. First, while examining ToM in people with and without ASD is a powerful way to test theoretically grounded links between ToM and social behaviour, further validation of the CarToM is required with other ecologically valid measures of social ability (e.g., observer-rated social skills, social network size) and ToM (e.g., video-based tasks, such as Brewer et al., 2017). Such investigations are particularly necessary beyond the study of autism and autistic traits, as autism is characterised by other atypical behaviours (e.g., repetitive and restricted behaviours) outside the social domain. Second, despite strengths in our sample characteristics, such as a similar number of male and female participants, which is rare in autism research, our findings require replication in independent and heterogeneous samples, both online and in-person. One intriguing finding in this study that requires further exploration is that CarToM performance (both ToM and non-ToM) was positively correlated with age. This should be followed up to determine whether it is a robust effect. Further studies will also enable exploration of CarToM task performance in relation to other socio-demographic variables not collected in this study (e.g., socio-economic status, ethnicity, gender identity that differs from sex). Third, it could be argued that humour appreciation, which the CarToM taps into, could differ as a function of autism or other individual differences. While this seems unlikely to explain our results, given that autism-related effects were specific to ToM cartoons only, future research should explore how differing humour appreciation is linked to autism, social cognition, and other individual differences more generally. Finally, although the Spot the Word task was specifically chosen to estimate general mental ability because of its similar task demands to the CarToM, it will be important to assess the CarToM against more detailed measures of both verbal and non-verbal intellectual ability.

In summary, using speed–accuracy scores, we found that the novel CarToM task is (1) sensitive to individual differences in ToM ability, (2) not confounded by general mental ability, (3) related to autistic traits across the (sub)clinical range, and (4) uniquely predictive of ToM difficulty in autistic adults. Future research is now required to test the CarToM’s relationship with other measures of “real-world” social ability. We suggest that the methods and analyses in this study could be fruitfully employed in numerous strands of research exploring mechanisms and individual differences associated with typical and atypical ToM ability, including both large-scale online data collection and in-depth neurocognitive studies. Together, the theoretically grounded CarToM will enable further progress in theory development, empirical research, and practices in the field of (a/typical) social cognition.

## Supplemental Material

sj-docx-1-qjp-10.1177_17470218231165251 – Supplemental material for Linearly integrating speed and accuracy to measure individual differences in theory of mind: Evidence from autistic and neurotypical adults Supplemental material, sj-docx-1-qjp-10.1177_17470218231165251 for Linearly integrating speed and accuracy to measure individual differences in theory of mind: Evidence from autistic and neurotypical adults by Lucy Anne Livingston, Punit Shah and Francesca Happé in Quarterly Journal of Experimental Psychology

sj-docx-2-qjp-10.1177_17470218231165251 – Supplemental material for Linearly integrating speed and accuracy to measure individual differences in theory of mind: Evidence from autistic and neurotypical adults Supplemental material, sj-docx-2-qjp-10.1177_17470218231165251 for Linearly integrating speed and accuracy to measure individual differences in theory of mind: Evidence from autistic and neurotypical adults by Lucy Anne Livingston, Punit Shah and Francesca Happé in Quarterly Journal of Experimental Psychology

## Notes

1 Effects sizes for non-parametric tests are rank biserial correlation coefficients.
