# Supplementary material for: Linearly integrating speed and accuracy to measure individual differences in theory of mind: Evidence from autistic and neurotypical adults
Source: Q J Exp Psychol (Hove). 2023 May 18;77(2):287–97. doi: 10.1177/17470218231165251 (PMC10798019; doi:10.1177/17470218231165251)
Supplement: sj-docx-1-qjp-10.1177_17470218231165251 – Supplemental material for Linearly integrating speed and accuracy to measure individual differences in theory of mind: Evidence from autistic and neurotypical adults [file sj-docx-1-qjp-10.1177_17470218231165251.docx]

**Supplementary Materials**

Linearly Integrating Speed and Accuracy to Measure Individual Differences in Theory of Mind: Evidence from Autistic and Neurotypical Adults

**Supplementary Figure 1**

*Distribution Plots by Group for ToM (a) and Non-ToM (b) Scores on the CarToM.*

####
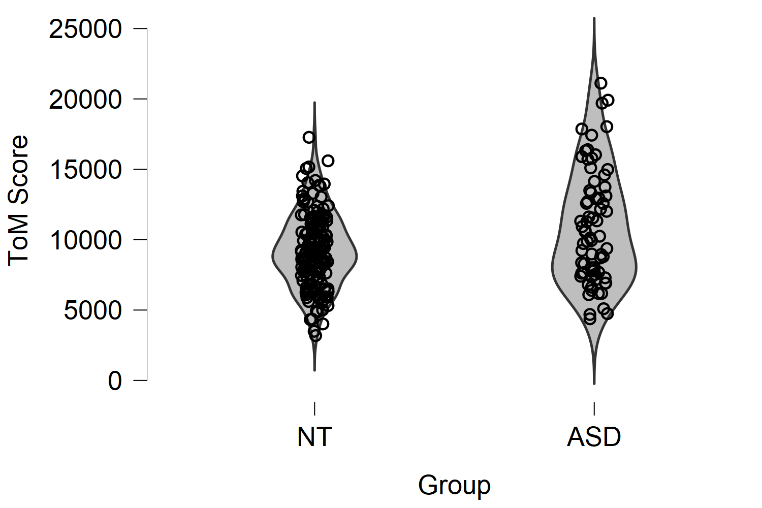


**a**

**b**

####
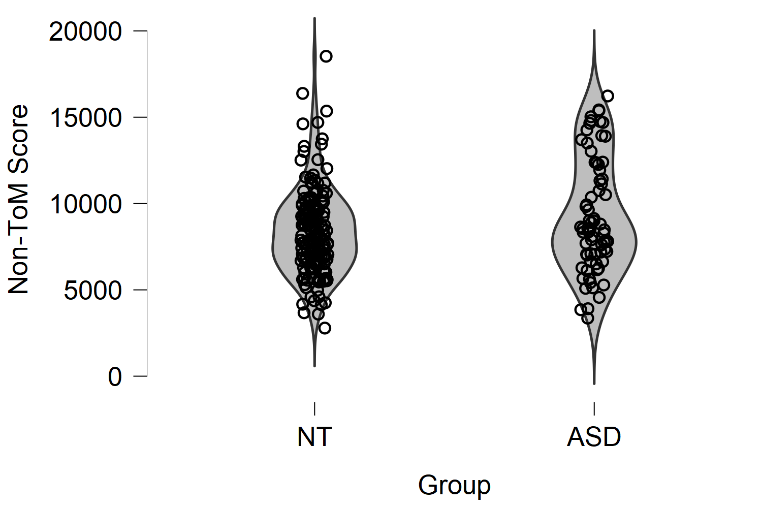


*Note.* NT, Neurotypical group; ASD, Autism Spectrum Disorder group. The y-axis represents Linear Integrated Speed-Accuracy Scores (Vandierendonck, 2017); greater scores reflect longer RTs in ms, corrected for accuracy, i.e., poorer performance.

**Supplementary Table 1**

*Mean LISAS Response Time and Accuracy Scores by Group for the CarToM and Other ToM Tests.*

|  | ASD (*n* = 72) | Neurotypical (*n* = 165) |
| --- | --- | --- |
|  | *M*(*SD*) | *M*(*SD*) |
| CarToM: ToM (LISAS)^a^ | 10872(4018) | 9265(2608) |
| CarToM: ToM Accuracy (%) | 82.44(15.49) | 90.13(9.68) |
| CarToM: ToM RT (ms)^b^ | 8724(3167) | 7995(2058) |
| CarToM: Non-ToM (LISAS)^a^ | 9296(3309) | 8329(2521) |
| CarToM: Non-ToM Accuracy (%) | 89.39(14.24) | 94.03(6.28) |
| CarToM: Non-ToM RT (ms)^b^ | 8151(2840) | 7537(2162) |
| RMET Accuracy (%) | 68.14(18.75) | 75.03(9.61) |
| Animations ToM Accuracy (%) | 49.64(34.18) | 63.28(29.80) |
| Animations Goal-directed Accuracy (%) | 68.21(28.53) | 69.53(24.55) |
| Animations Random Accuracy (%) | 82.50(24.58) | 84.22(18.94) |

*Note.* CarToM, Cartoons Theory of Mind task; RMET, Reading the Mind in the Eyes Test (Baron-Cohen et al., 2001a); Animations, Frith-Happé Animations Test (White et al., 2011); RT, response time. ^a^Linear Integrated Speed-Accuracy Scores (Vandierendonck, 2017); greater scores reflect longer RTs (ms), corrected for accuracy, i.e., poorer performance. ^b^Mean RTs for correct trials, which excludes trials that were ± 3 *SD*s of each participant’s overall mean RT.

**Supplementary Table 2**

*Correlations Between all Measured Variables.*

|  | 1 | 2 | 3 | 4 | 5 | 6 | 7 |
| --- | --- | --- | --- | --- | --- | --- | --- |
| 1 – CarToM: ToM (LISAS) | - |  |  |  |  |  |  |
| 2 – CarToM: Non-ToM (LISAS) | .79*** | - |  |  |  |  |  |
| 3 – Group (1 = ASD, 0 = NT) | .23*** | .16* | - |  |  |  |  |
| 4 – Autistic Traits | .20** | .08 | .77*** | - |  |  |  |
| 5 – Age | .17** | .17** | .11 | .19** | - |  |  |
| 6 – Sex (1 = Male, 0 = Female) | .01 | -.03 | -.01 | .10 | .19** | - |  |
| 7 – General Mental Ability (LISAS) | .18** | .20** | -.04 | -.03 | .15* | -.09 | - |

*Note.* NT, Neurotypical; ASD, Autism Spectrum Disorder. CarToM and General Mental Ability scores are Linear Integrated Speed-Accuracy Scores (LISAS; Vandierendonck, 2017); greater scores reflect longer RTs (ms), corrected for accuracy, i.e., poorer performance. Effects sizes for binary variables are rank biserial correlation coefficients. ****p* < .001, ***p* < .01, **p* < .05.
